# Nano and chelated iron fertilization influences marketable yield, phytochemical properties, and antioxidant capacity of tomatoes

**DOI:** 10.1371/journal.pone.0294033

**Published:** 2023-11-08

**Authors:** Arifur Rahman, Thomas Harker, Wayne Lewis, Khandakar Rafiq Islam

**Affiliations:** The Ohio State University South Centers, Piketon, OH, United States of America; Bahauddin Zakariya University, PAKISTAN

## Abstract

Iron (Fe) is one of the limiting micronutrients essential for crop productivity. The goal of our study was to evaluate the effects of different sources and rates of Fe fertilization on the marketable yield, physical and chemical properties, and phytochemical quality of fresh market tomatoes (*Solanum Lycopersicum L*., cv. Sunbrite). A factorial experiment under a drip-irrigated plasticulture system was conducted in a completely randomized design with two sources of Fe (nano vs. chelated) and four rates of application (0, 10, 20, and 40 mg/L) with four replications. Results indicated that relative chlorophyll concentration in the leaf (SPAD index) increased significantly (by 24 to 27%) with 10 and 20 mg/L of both nano- and chelated Fe fertilization compared to the control. Increasing Fe fertilization decreased the leaf SPAD readings. The total fruit yield of tomato was 1.6 to 1.8 times higher under the chelated- and nano Fe fertilization and the increase in yield was significantly higher under the chelated Fe fertilization, when compared to the control. In contrast, the tomato harvest index was highest under 10 and 20 mg/L of nano Fe than under other Fe treatments. While the chelated Fe fertilized tomatoes had significantly higher concentrations of vitamin C (34%), ß-carotene (6%), total carotene (25%), flavonoid (17%), and polyphenol (66%), the nano Fe, in contrast, increased ß-carotene, total carotene, and polyphenol concentrations by 25, 33, 51, and 7%, respectively, compared to the control. The 20 mg/L chelated Fe significantly increased the vitamin C, total carotene, flavonoid, polyphenol concentration, and antioxidant capacity more than any other Fe treatments. Based on the principal components analyses, vitamin C, lycopene, and anthocyanin were identified as the core indicators of the tomato nutrition quality index (*NQ*_*Index*_). The *NQ*_*Index*_ ranged from 47 to 54, falling within the medium level of nutritional quality (*40 to <70)*. In conclusion, the chelated Fe, when applied at 20 mg/L, was the most appropriate rate based on highly correlated connectivity for the phytochemicals syntheses associated with the improved tomato antioxidant capacity.

## Introduction

Tomato is a popular high-value specialty crop with a global average production of 37.1 Mg/ha [1]; however, imbalanced nutrient management practices and climate change can impact tomato production and quality [2]. Tomato is an important source of phenolic compounds, carotenoids, vitamins, and nutrients that are associated with public health benefits [3]. To overcome the increasing demand for high-quality tomatoes, existing chemical fertilization needs to be supplemented with adequate and balanced nutrition, especially micronutrients. Micronutrients are critically essential to regulate biochemical and enzymatic functions associated with crop yield and produce quality [4].

Fe is the third most limiting essential micronutrient for plant productivity [5, 6]. Due to its dynamic oxidation-reduction properties, Fe plays a critical role in plant physiological and biochemical pathways such as DNA synthesis, respiration, and photosynthesis processes. Moreover, Fe is a critical component of several vital enzymes that carry out electron and oxygen transport functions, facilitate chemical transitions, and regulate protein stability, and thus are required for a wide range of metabolic functions in plants [4, 6, 7]. Being the fourth most abundant chemical element in the lithosphere, total Fe content is high in most soils; however, its availability to plants is limited. While abundant in most soils, the ionic activity of Fe is low as it often reacts with clay minerals and forms insoluble Fe compounds [6]. In contrast, if Fe is taken up by plants in excessive amounts, it becomes toxic and acts catalytically via the Fenton reaction to generate reactive oxygen-based radicals that can damage vital cellular constituents of plants by lipid peroxidation [6]. Iron deficiency is a common nutritional disorder in many crops, resulting in poor yields and reduced nutritional quality [6, 8] An imbalance among input, solubility, and demand are the primary causes of Fe deficiency in crops, most notably specialty crops and vegetables. Therefore, the Fe nutrition of plants must respond to Fe stress between Fe deficiency and overloading by optimizing Fe sources and rates.

Fe fertilization is a new direction for global agricultural production as a supplement to Fe deficiency in crops and vegetables [9]. Currently, various types of Fe compounds, including Fe sulfate, Fe oxide, or Fe chelates are commonly used. Among these compounds, Fe chelates are the most common and have shown higher absorption rates when compared to the conventional mixed Fe fertilizers [10]. Chelated nutrients are protected from chemical oxidation, precipitation, biodegradation, and immobilization under certain conditions because the ligand (organic molecule) can combine and form rings encircling the micronutrients [11]. Chelates are, by nature, very soluble in water, but only slightly dissociated. The gradual release of chelated micronutrients, such as Fe, increases their absorption by plants and prevents their excessive uptake. The pincer-like way the micronutrient is bonded to the ligand alters the surface properties of the chelated Fe, enhancing its uptake efficiency by the plants. The effectiveness of Fe, Mn, and Zn with traditional or novel chelates and neutral complexing agents has been investigated for crop nutrition and production [12, 13]. Several studies, in contrast, have reported that chelated micronutrients, especially Fe, have limitations associated with their application and plant absorption [14]. It is reported that at a pH above 6, almost 50% of the chelated Fe becomes unavailable to plants.

In recent years, nanotechnology has become increasingly adapted in agriculture, aiming to improve fertilizer-use efficiency, minimize losses, and increase farm economics through precision nutrient management practices [15]. It is reported that Fe nanomaterials (1 to 100 nm) have excellent fertilizing properties due to their smaller size, faster reactivity, and ability to interact with biological systems to enhance the growth, development, and stress tolerance of plants [9, 16, 17]. Several studies have reported that Fe nanoparticles are more effective in increasing Fe availability to growing plants compared to the commonly used Fe fertilizers or chelated Fe fertilization [9]. Therefore, substituting nano Fe fertilizer for conventional or chelated Fe fertilizer is expected to improve Fe availability in a controlled and efficient manner to increase plant growth, yield, and bioactive phytochemicals concentration.

Bioactive phytochemicals, including flavonoids, phenolic acids, alkaloids, and carotenoids are commercially valued products due to their wide array of applications in the medical, cosmetic, agriculture, and food industries [18]. Epidemiological studies on tomatoes have revealed their capacity to reduce the risk of certain types of diseases and balance human nutrition [19]. Despite an improvement in tomato growth and yield by Fe fertilization, information on bioactive phytochemicals as influenced by nano and chelated Fe fertilization is limited. The objectives of our research were to (1) evaluate and compare the effects of variable rates of nano and chelated Fe fertilization on the growth and yield of fresh-market tomatoes under a drip-irrigated plasticulture system; (2) determine physical and chemical properties and bioactive phytochemical compounds in tomato fruits; and (3) perform principal components analyses to ascertain Fe fertilization effects on nutritional quality associated with antioxidant capacity of tomatoes.

## Materials and methods

### Study site

The study was conducted at The Ohio State University South Centers in Piketon, Ohio, USA (lat. 39.07° N, long. 83.01° W with mean sea level elevation of 103 m). While the average monthly maximum air temperature of 32.2°C was recorded as highest in August, it was lowest at 15.6°C in September. Mean annual rainfall is 96.2 cm, with about 55% of the precipitation falling during the crop-growing season (May to September). The highest monthly rainfall (15 cm) was recorded in July. The monthly relative humidity ranges from 79 to 93%, soil temperatures at 15 cm deep range between 3 and 30°C, and solar radiation ranges between 9,980 to 43,000 KW/m^2^. The soil is a poorly drained Doles silt loam [20] with a pH of 6.0±0.3, total organic carbon of 0.82±0.23%, total nitrogen of 0.105±0.024%, bulk density of 1.28±0.04 g/cm^3^, and sand, silt, and clay of 30±4, 55±2, and 15±2%, respectively, at 0 to 20 cm depth.

### Experiment and cultural practices

A factorial experiment (2 Fe sources x 4 rates of application) in a completely randomized design was conducted. Both nano and chelated Fe were fertilized at rates of 0 (control), 10, 20, and 40 mg Fe/L of water via drip irrigation. The Fe treatments were replicated four times and each replicated plot was 2 m wide x 5 m long with a 50 cm buffer between plots. The nano Fe (Fe_2_O_3_) material was collected from the Aqua-Yield^®^ Company (Sandy, UT, USA) and the chelated Fe (iron-Ethylene di-amine di[o-hydroxyphenylacetic acid, FeEDDHA]) was collected from the Miller^®^ Chemical and Fertilizer, LLC (Hanover, PA, USA)

Fresh market tomato (cv. Sunbrite) was seeded into 72-cell plug plastic trays containing Metro Mix 360^®^ soilless media and placed in the controlled plant growth chamber for germination. After germination, about 25 to 30 cm tall seedlings were planted in the field during the third week of May 2021. Prior to laying plastic, the field was chisel plowed followed by surface application of a basal dose of NPK 19-19-19 fertilizers at the rate of 100 kg/ha. Plastic rows were 1.6 m apart with tomato seedlings being planted and spaced 60 cm apart within rows of raised beds using a waterwheel transplanter.

Nano and chelated Fe treatments were applied twice on tomato plants in mid-June and early July (maximum vegetative growth stage). The Fe treatments and watering of the tomato plants were applied via drip irrigation emitters that were inserted into a soil depth of 10 cm. Irrigation was applied as required based on 75% of the maximum allowable depletion of available soil moisture. All irrigation valves were shut off except for the Fe treatment that was being applied. Drip lines were pressurized, and the respective Fe treatments were injected into the irrigation. Each time, the Fe treatments took almost 15 min to inject and then were allowed to irrigate for 10 more min to purge the lines, then the valve was shut off at each treatment. The header line was then uncapped to empty the header line between each treatment. Cultural practices and fungicides were applied following standard recommendations from the Midwest Vegetable Production Guide for Commercial Growers (ID-56).

### Growth and yield parameters of tomato

At various growth stages of tomato plants, leaf SPAD readings, as an indicator of relative chlorophyll concentration and nitrogen uptake, were determined using the Minolta^®^ SPAD meter. After each harvest, all the tomato fruits were graded and sized as small, medium, or large, and their respective numbers and weights were recorded. The data were processed to calculate the growth and yield parameters of tomatoes including harvest index (fruit yield/biomass and fruit yield).

### Color index, soluble solids, titratable acidity, and taste index of tomato

Immediately following harvest in the state of edible maturity at peak season, randomly selected ripened fresh tomatoes from each replication were washed with water to remove adhered soil particles followed by rubbing with soft tissue paper to dry prior to performing any measurements. Tomato color index was measured at 10 points around the equatorial region on the clean whole tomato surface [21] using a handheld Minolta^®^ CR-200 Chroma meter (Minolta Camera Co., Osaka, Japan). After color index measurement, the tomatoes were preserved in a refrigerator (-20°C) until the extraction of juice. The lyophilized tomatoes were cut into small pieces and then blended until solids were tuned into juice and sieved (1 mm mesh) to ensure a uniform mixture.

Soluble solid (°Brix), as a measure of total sugar content, was determined by refractometry using an ATAGO^®^ digital refractometer [22]. Two drops of tomato juice were placed on the prism of the equipment surface, and the percentage of soluble solids was shown directly, expressed in terms of °Brix. Titratable acidity (TA) was determined by titrating 10 g of a homogenized sample of tomato juice. After dilution with 50 mL of distilled deionized water, the solution was titrated against 0.1% NaOH solution using a phenolphthalein indicator at a pH of 8.2 [23], and the result was reported as a percentage. The taste or sweetness index (TI) of tomato juice was calculated [24, 25] as follows:

$$TI=\frac{SS}{20TA}+TA$$ (1)

### Vitamin C, lycopene, and carotenes of tomato

To measure vitamin C [26], a 10 mL sample of tomato juice was taken in a 50 mL volumetric flask followed by the addition of 25 mL of 5% metaphosphoric acid and 10% glacial acetic acid mixture and gently shaken for 2 hr. The upper-layered liquid was carefully transferred into a 50 mL volumetric flask and diluted up to the mark using a 5% metaphosphoric acid and 10% glacial acetic acid mixture followed by centrifugation at 4,000 rpm for 10 min and then filtered. A 4 mL sample of the supernatant was taken in a 15 mL plastic test tube and 0.5 ml of 3% of bromine water was added to oxidize the ascorbic acid in the supernatant to dehydroascorbic acid, followed by the addition of 0.5 mL of 10% thiourea solution to remove excess bromine. Then 1 mL of 2,4-DNPH solution was added to form osazone. All the standards, samples, and blank solution were kept at 37°C temp. for 3 hr in a thermostatic water bath, followed by cooling in an ice bath for 30 min, and then treated with 5 mL of 85% chilled sulfuric acid with constant stirring. Finally, the absorbance of the colored solution was measured at 521 nm wavelength using a Shimadzu^®^ UV-Visible Spectrophotometer.

The lycopene and β-carotenes were measured by following Nagata and Yamashita (1992) [27]. A 1.0 g sample of tomato juice was taken in a test tube and 10 mL of freshly prepared acetone: hexane (4:6) mixture was added into the test tube followed by homogenizing the mixture using a shaker for 10 min. The absorbance (A) of the homogenized mixture was measured at 663, 645, 505, and 453 nm using the Shimadzu^®^ spectrophotometer, and the values of lycopene and β-carotene were calculated as follows:

$$Lycopene\;(mg/100\;mL)=0.0485A_{663}+0.204A_{645}+0.372A_{505}‐0.0806A_{453}$$ (2)

$$\beta ‐carotene\;(mg/100\;mL)=0.216A_{663}‐1.22A_{645}‐0.304A_{505}+0.452A_{453}$$ (3)

To measure total carotene [27], the optical density of the homogenized mixture as described above was measured at 663.6 nm for chlorophyll *a* and 646.6 nm for chlorophyll *b* and 470 nm using the Shimadzu^®^ spectrophotometer as follows:

$$Chlorophyll\;a\;(\mu g/mL)=12.25A_{663.6}‐2.25A_{646.6}$$ (4)

$$Chlorophyll\;b\;(\mu g/mL)=20.31A_{646.}6–4.91A_{663.6}$$ (5)

$$Total\;carotene\;(\mu g/mL)=[1000A_{470}‐2.27\;(Chl\;a)‐81.4\;(Chl\;b)]/227$$ (6)

### Flavonoid, polyphenol, anthocyanin, and antioxidant capacity of tomato

A 2.0 g sample of tomato juice was taken in 15 mL plastic centrifuge tubes and 5 mL of methanol:water:formic acid mixture (at 70:30:0.1) was added [28], then the mixture was shaken for 10 min, ultrasonicated for 10 min, and centrifuged at 5,000 rpm for 10 min. The supernatant liquid was separated and transferred to amber-colored sample vials placed in the dark. This process was repeated three more times and the extracted volumes were added together to determine antioxidants in tomatoes.

Total flavonoid was determined spectrophotometrically [29]. A 1 mL extracted sample was taken in a 15 mL plastic tube followed by the addition of 4 mL distilled deionized water, and the mixture was homogenized using a vortex. After homogenization, 0.33 mL of 5% sodium nitrite (w/v) and 0.3 mL of 10% aluminum chloride (w/v) were added to the solution. The sample was allowed to stand for 5 min after the addition of each reagent. Finally, 2 mL of 1M NaOH solution was added to the mixture and the volume was made up to 10 mL with distilled deionized water. The absorbance of the pink-colored mixture was measured with a Shimadzu^®^ UV-Visible Spectrophotometer. Four extraction cycles were used to reach 100% recovery of the total flavonoids from each sample. The results were expressed as catechin equivalents per mg/100 g dry weight.

Total polyphenols were analyzed spectrophotometrically by following the procedure of Rapisarda et al. [30]. A 1 mL sample of extract was taken in a 15 mL plastic tube. Then 6 mL of distilled deionized water and Folin-Ciocalteu reagent were added and allowed to react for 5 min. After that, 2 mL of 20% Na_2_CO_3_ (w/v) solution was added and heated at a mixture of 40°C for 2 min. The blue-colored mixture’s absorbance was measured at 760 nm with a UV-visible spectrophotometer (Biomate 3S, Thermo Scientific). Four extraction cycles were used to reach 100% recovery of the total polyphenols from each sample. The results were expressed as (mg gallic acid/g dry weight).

Total anthocyanin was analyzed following the pH differential methodology with spectrophotometry [30]. An amount of 1.0 g lyophilized sample was taken in a 15 mL centrifuge tube and then 5 mL of pH 1.0 buffered solution (0.2 M KCL and 0.2M HCl) was added, followed by vortex for 5 min. The mixture was then ultrasonicated for 5 min followed by centrifugation at 5,000 rpm for 10 min. The supernatant was transferred into amber-colored vials and kept in the dark. This procedure was repeated three more times and the mixture was brought to a desired volume using pH 1.0 buffer solution. Similarly, another extraction was carried out using a buffer with pH 4.5 (1M CH_3_COONa and 1M HCl). Absorbance (A) of the mixture was determined using a UV-visible spectrophotometer at two different wavelengths (520 and 700 nm). Total anthocyanin was calculated using the following equations:

$$Absorbance=(A_{520}‐A_{700})pH1‐(A_{520}‐A_{700})pH\;4.5$$ (7)

$$Anthocyanin\;\;(\frac{mg}{g})=\frac{Abs\;x\;MW\;x\;DF\;x\;1000}{\varepsilon \;x\;1\;cm\;UV\;cell\;x\;amount\;of\;tomato}$$ (8)

Where MW is molecular weight (449.2 g) and ε is 26,900. The results were expressed as mg of cyanidin-3-glucoside chloride per g of dry weight.

The antioxidant capacity of tomatoes was measured by the ferric-reducing power method [31]. A 1.0 mL sample of extract along with 2.5 mL of phosphate buffer at pH 6.6 and 2.5 mL of 1% potassium ferrocyanide solution was taken into a 15 mL test tube. The mixture was homogenized using a vortex followed by incubation at 50°C for 20 min. After incubation, 2.5 mL of 10% trichloroacetic acid was added along with 2.5 mL of water and 0.5 mL of 1% FeCl_3_ solution. The mixture was homogenized again using a vortex and was allowed to stand for 30 min in the dark. The green color of the mixture was measured at 700 nm using a Shimadzu^®^ UV-visible Spectrophotometer. Results were expressed as μM equivalent Trolox per g of dry weight.

### Nutritional quality index of tomato

The nutritional quality index *(NQ*_*Index*_*)* of tomato was calculated based on its antioxidant properties as reported by Frusciante et al. [32]:

$$Copt=C_{a}+[0.5\;x\;C_{a}]$$ (9)

Where *Copt* is the optimal concentration and Ca is the average concentration of antioxidant parameters, which were obtained from the literature. By modifying the above equation, the *NQ*_*Index*_ was calculated as follows:

$$NQ_{Index}=[\;(C_{x}\;K_{x})/Copt]$$ (10)

Where C_x_ is the concentration of the antioxidant (x) parameters in the sample, *Copt* is the optimal concentration of the component, and K_x_ is the coefficient of relative weight of the component (K = 15 for vitamin C; K = 20 for lycopene; K = 10 for β-carotene; K = 15 for flavonoid; K = 10 for total polyphenol; and K = 15 for total anthocyanin) as per Frusciante et al. [32]. The K values were adopted based on the importance of the antioxidants used in the diet for better public health.

To avoid any bias and data redundancy for calculating the *NQ*_*Index*_ based on K coefficients, we proposed a new approach to calculate the *NQ*_*Index*_ using a minimum data set (MDS) based on principal components analyses (PCA). All the measured antioxidant data of tomato fruits were included in the PCA model using OriginPro^®^. The PCs that had eigenvalue > 1.0 and explained at least 5% of the variation in the data were selected as MDS. Based on the PCA, vitamin C was grouped in PC1, lycopene in PC2, and total anthocyanin in PC3, and were selected based on their highest weighted eigenvector values and also the importance of antioxidants in the PCA group. Therefore, about (3/6 = 50%) of the data was selected as MDS for the *NQ*_*Index*_ calculation:

$$NQ_{Index}=[Weight\;of\;\;(PC1)*vitamin‐C]+[Weight\;of\;\;(PC2)*lycopene]+[Weight\;of\;\;(PC3)*anthocyanin]$$ (11)

$$Where,\;Weight\;of\;PC=\frac{Variance\;\;(\%)\;of\;individual\;PC}{Total\;variance\;\;(\%)}$$ (12)

### Quality analysis / Quality control

All the analytical precision as determined by Quality analysis (QC) / Quality control (QC) procedures, reagent blanks, and internal standards, was better than ±10%. Distilled deionized water obtained through a Milli-Q water purification system was used. The standards, fine analytical grade solvents, and reagents used were obtained from Sigma-Aldrich^®^ (St. Louis, MO, USA).

### Statistical analysis

Data on growth, marketable yield, physicochemical properties, antioxidants, and nutritional quality of tomatoes as dependent variables, in response to different rates of nano and chelated Fe fertilization, were processed for multivariate statistical analysis using the SAS^®^ [33]. Both nano and chelated Fe levels were considered as fixed predictor variables. Data were subjected to a two-way analysis of variance procedure. Means of simple and interactive effects of predictor variables (Fe source, Fe rate, and Fe source x rate) on tomato growth and yield parameters were separated by the Duncan Multiple Range Test (DMRT) at p ≤ 0.05 unless otherwise mentioned. Principal Components Analyses and correlation matrix were performed to ascertain the relationship between the phytochemical properties of tomatoes with their antioxidant capacity using OriginPro^®^.

## Results

### Growth, yield, and physicochemical properties of tomato

Leaf SPAD reading, as a relative measure of chlorophyll concentration, was influenced by Fe fertilization without any Fe source x rate interactions (**Table 1**). Both nano and chelated Fe significantly increased the SPAD readings by 27% and 24%, respectively, when compared to the control. The effect on leaf SPAD was more pronounced at 10 and 20 mg Fe/L of both nano and chelated Fe treatments. Averaged across the Fe sources, increasing rates of Fe of fertilization decreased leaf SPAD reading.

**Table 1:** Effects of different rates of nano and chelated Fe fertilization on the growth and fruit yield of fresh market tomatoes under a drip-irrigated plasticulture system.

Iron	Rate	SPAD	Marketable fruit yield (Mg/ha)				Harvest
source	(mg/L)	reading	Small	Medium	Large	Total	index
Control		21.5y^≠^	17.2z	10.5z	3.0z	30.7z	0.71y
Nano Fe		27.2x	26.7x	20.8x	7.4y	54.9x	0.81x
Chelated Fe		26.6x	22.7y	18.0y	9.1x	49.9y	0.75y
**Fe source x rate**							
Nano Fe	10	28.4	25.3	22.9	9.6	57.8	0.83
	20	27.3	29.6	21.8	5.2	56.6	0.81
	40	26.0	25.1	17.7	7.5	50.4	0.79
Chelated Fe	10	28.7	23.0	18.1	9.3	50.4	0.76
	20	25.8	23.2	18.3	9.3	50.8	0.74
	40	25.3	22.2	17.6	8.6	48.6	0.75
Probability (>F)		0.28	0.51	0.12	0.43	0.34	0.29

^≠^Means under each column separated by the same lowercase letter (x, y, and z) were not significantly different among the iron sources at p≤0.05.

Both nano and chelated Fe significantly influenced the size, fruit yield, and harvest index of tomatoes without a Fe source x rate interaction (**Table 1**). The amount of smaller-sized tomato yield was significantly higher in the nano Fe treatments followed by the chelated Fe when compared to the control; however, the control had the highest percentage of smaller-sized tomatoes. Nano Fe fertilization, when applied at 10 mg/L, resulted in the highest production of large-sized tomatoes. Like nano Fe, the chelated Fe fertilization increased large-sized tomato production. When combined, the total yield of tomato was 1.6 to 1.8 folds higher under chelated and nano Fe fertilization compared to the control and the difference in total yields between chelated and nano Fe treatments was significant. Regardless of the Fe source, higher rates of Fe fertilization decreased tomato production. Nano Fe significantly increased the tomato harvest index by 8 to 14% when compared to both chelated Fe and control treatments. Tomato harvest index was highest under 10 and 20 mg/L of nano Fe than that of other Fe treatments.

Tomato physicochemical properties are variably influenced by nano and chelated Fe fertilization compared to the control without any Fe source x rate interactions, except for sugar concentration (**Table 2**). While the color index of tomatoes was not significantly varied by Fe sources, increasing rates of chelated Fe fertilization (40 mg/L) significantly decreased the color index. Both nano and chelated Fe fertilization marginally affected the tomato juice pH when compared to the control. The titratable acidity was significantly higher (0.60%) under chelated Fe when compared to both nano Fe (0.55%) and the control (0.53%) treatments. In contrast, sugar concentration was significantly higher (5.3%) under the control than that of both nano and chelated Fe fertilization (5.1%). While increasing rates of chelated Fe fertilization decreased sugar concentration, in contrast nano fertilization increased sugar concentration in tomatoes. The sugar:acid was decreased more by chelated Fe with respect to the control. Increasing rates of Fe fertilization asymmetrically deceased sugar:acid, regardless of the Fe sources. Likewise, the taste index of tomatoes was significantly decreased more by chelated Fe fertilization than that of nano Fe when compared to the control.

**Table 2:** Effects of different rates of nano and chelated Fe fertilization on color, pH, titratable acidity, soluble solids concentration, and taste index of fresh market tomatoes under a drip-irrigated plasticulture system.

Iron	Rate	Color	pH	Acidity	Sugar	Sugar:	Taste index
source	(mg/L)	index		(%)	(g/100 g)	acid	(TI)
Control		34.1x^≠^	4.4x	0.53y	5.3x	10.1x	3.2x
Nano Fe		34.5x	4.3y	0.55y	5.1y	9.4xy	3.0xy
Chelated Fe		34.7x	4.3y	0.60x	5.1y	8.5y	2.8y
**Fe source x rate**							
Nano Fe	10	35.0	4.3	0.53	5.2	9.7	3.0
	20	32.4	4.3	0.56	5.6	10	3.4
	40	32.8	4.3	0.55	4.6	8.5	2.5
Chelated Fe	10	34.0	4.3	0.56	4.8	8.6	2.6
	20	39.2	4.3	0.57	5.2	9.1	2.9
	40	31.0	4.3	0.68	5.3	7.8	2.9
Probability (>F)		0.23	0.91	0.10	<0.001	0.34	0.14

^≠^Means under each column separated by the same lowercase letter (x, y, and z) were not significantly different among the mean values of different iron sources at p≤0.05.

* Indicates significant interaction of Fe source x rate at p≤0.05.

### Antioxidant properties and nutritional quality of tomato

Both nano and chelated Fe fertilization have increased antioxidant properties with an interactive effect of Fe source x rate on total carotene, polyphenol, and flavonoid concentrations of tomatoes **(Table 3)**. Chelated Fe fertilization significantly increased vitamin C (by 34%), ß-carotene (by 6%), total carotene (by 25%), flavonoid (by 17%), and polyphenol (by 66%) concentrations when compared to the control (**Table 3**). In contrast, the nano Fe fertilization only increased ß-carotene, total carotene, and polyphenol concentrations by 25, 33, 51, and 7%, respectively, than that of the control. Chelated Fe had a significantly higher concentration of vitamin C (by 25%) and flavonoid (by 17%), but a lower concentration of total carotene (by 6%) than that of the nano Fe fertilization. When applied at 20 mg/L, chelated Fe significantly increased the vitamin C, total carotene, flavonoid, and polyphenol concentration more than all other Fe treatments. Both chelated and nano Fe, when applied at 20 mg/L, resulted in a similar anthocyanin concentration in tomatoes. The Fe source x rate significantly affected the concentration of total carotene, flavonoids, and polyphenols. The antioxidant capacity of tomatoes significantly increased with both sources of Fe fertilization (1.66 μM/g in nano Fe and 1.68 μM/g in chelated Fe), which were significantly higher (by 7 to 8%) than the control (1.55 μM/g).

**Table 3:** Effects of different rates of nano and chelated Fe fertilization on vitamin C, ß- and total carotenoids, flavonoid, polyphenol, and anthocyanin concentrations, and antioxidant activity of fresh market tomatoes under a drip-irrigated plasticulture system.

Iron	Rate	Vitamin C	Lycopene	β-carotene	Tot.-carotene	Tot.-polyphenol	Flavonoid	Tot.-anthocyanin	Antioxidant activity
source	(mg/L)				(mg/100 g)			(mg/g)	(μM/g)
Control		14.8y^≠^	6.3x	0.51y	1.11z	1.36y	35.4y	0.43x	1.55y
Nano Fe		15.8y	6.3x	0.64x	1.48x	2.06x	35.6y	0.46x	1.66x
Chelated Fe		19.8x	6.3x	0.65x	1.39y	2.26x	41.5x	0.49x	1.68x
**Fe source x rate**									
Nano Fe	10	16.5	6.2	0.64	1.42	2.10	33.1	0.46	1.57
	20	14.4	6.3	0.63	1.50	2.17	28.8	0.46	1.66
	40	16.5	6.3	0.65	1.50	1.92	45.0	0.46	1.64
Chelated Fe	10	18.9	6.3	0.64	0.94	1.61	42.3	0.51	1.66
	20	22.1	6.2	0.63	1.59	2.95	44.1	0.46	1.69
	40	18.3	6.4	0.69	1.64	2.23	38.3	0.49	1.70
Probability (>F)		ns	1.00	0.45	0.001	0.03	0.01	0.29	0.98

^≠^Means under each column separated by the same lowercase letter (x, y, and z) were not significantly different among the iron sources at p≤0.05.

* Indicates significant interaction at p≤0.05.

Among the six antioxidants of tomatoes used to calculate the *NQ*_*Index*_, the K coefficient values for the targeted antioxidants were chosen by considering the importance of those for public health benefits (**Table 4**). It was observed that the optimal concentration for the antioxidants was higher than the average concentration of tomatoes produced in response to Fe fertilization. However, the optimal concentration was between the cited literature’s values (**Table 4**).

**Table 4:** Literature cited concentration, average concentration, calculated optimal concentration, and coefficient of antioxidant compounds in tomato fruits. [32]*, [34]^¥^, [35]^≠^, [36]^≠^, [37]^¥^, [38]**.

Antioxidants	Literature cited	Average conc.	Optimal conc.	Coefficient
	conc.	used	(calculated)	(K)
		mg/100 g (fresh-weight)		
Vitamin-C	2.20–21*	10.5	15.75	15
Lycopene	1.86–14.62*	7.31	10.97	20
β-carotene	0.11–1.07*	0.54	0.80	10
Flavonoid	1.15–8.16*	4.08	6.12	15
Polyphenol	23.69–64.6^≠¥^	48.0	48.45	15
Anthocyanin	40–140**	70	105.0	15

**Fig 1:**
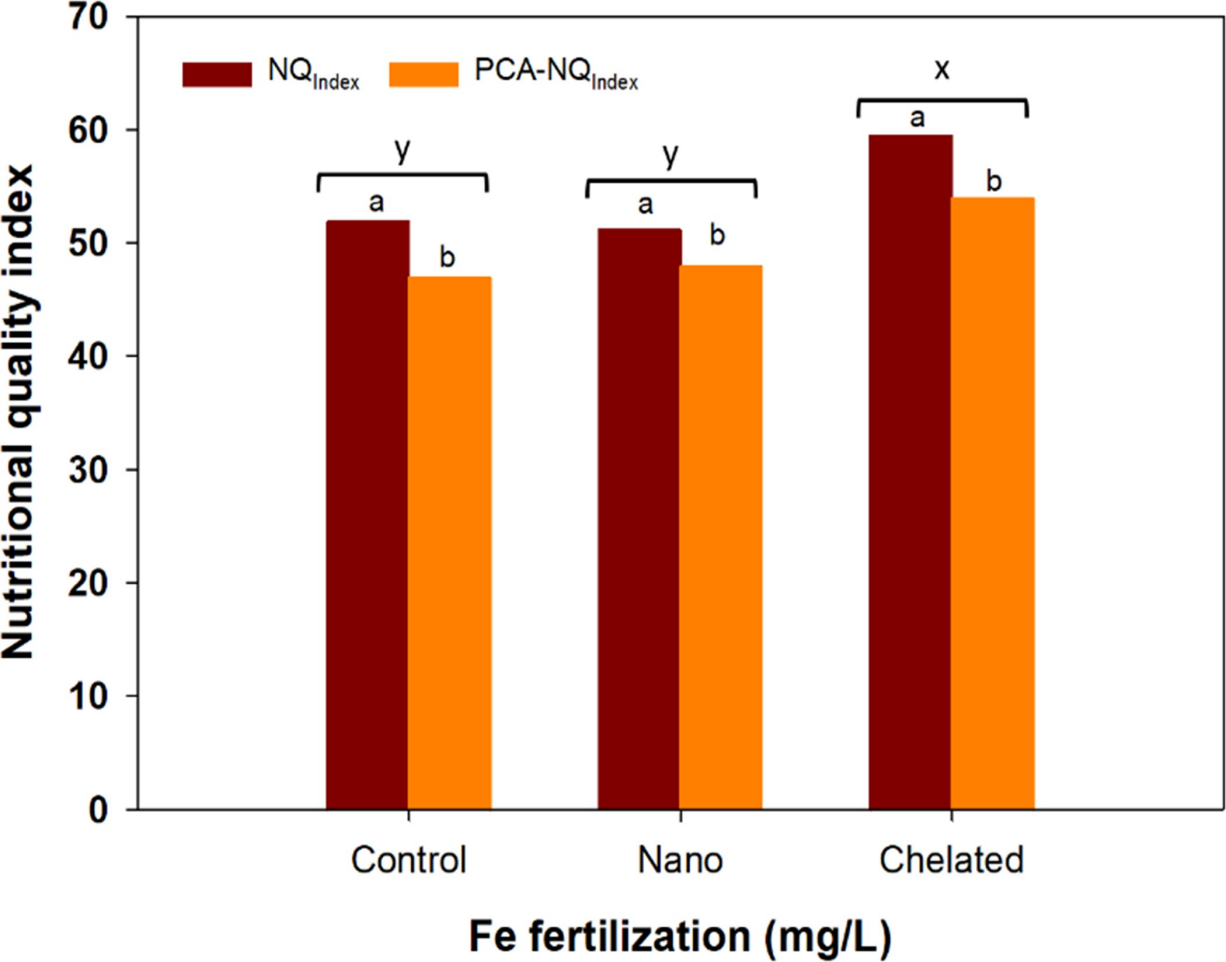
Nutritional quality of tomato, based on antioxidant properties, in response to nano and chelated Fe fertilization under a drip-irrigated plasticulture system [while the letters (a vs. b) indicated a significant difference between nutritional quality indices (NQ_Index_ vs. PCA-NQ_Index_) at p≤0.05, the letters (x vs. y) indicated a significant difference among Fe sources at p≤0.05].

Based on the standard approach [32], the scores of antioxidants for calculating the *NQ*_*Index*_ of tomatoes showed that the vitamin C had the highest values, followed by lycopene, polyphenol, β-carotene, anthocyanin, and flavonoids, respectively (**Table 5**). The chelated Fe had significantly higher scores for all antioxidants except lycopene when compared with nano Fe and the control treatments. The calculated *NQ*_*Index*_ values, based on the scores, were divided into three categories: (i) high antioxidant quality (*NQ*_*Index*_ > 70), (ii) medium antioxidant quality (*NQ*_*Index*_ < 70–40), and (iii) low antioxidant quality (*NQ*_*Index*_ < 40). It showed that the higher *NQ*_*Index*_ (63) values of tomatoes were achieved with 20 mg/L chelated Fe fertilization when compared to the lowest *NQ*_*Index*_ (47.5) values found with the 20 mg/L nano Fe fertilization (**Fig 1**). However, all the Fe treatments produced the medium level of *NQ*_*Index*_ values of tomatoes, which varied from 47.5 to 63. Averaged across the Fe rates, chelated Fe-treated tomatoes had the highest *NQ*_*Index*_ (59.5) values when compared to nano Fe-treated (48) and the control (47) tomatoes.

**Table 5:** Effects of different rates of nano and chelated Fe fertilization on antioxidant index scores of tomatoes under a drip-irrigated plasticulture system.

Iron	Rate	Vitamin C	Lycopene	β-carotene	Flavonoid	Polyphenol	Anthocyanin
source	(mg/L)				Index score		
	
Control		16.0y^≠^	11.4x	6.4y	4.5y	7.3y	6.2y
Nano Fe		12.6z	11.5x	8.5x	4.7y	7.4y	6.6z
Chelated Fe		18.8x	11.5x	8.1x	5.5x	8.6x	7.0x
**Fe source x rate**							
Nano Fe	10	15.7	11.4	9.4	5.3	6.8	6.6
	20	10.8	11.5	7.8	4.8	6.0	6.6
	40	11.3	11.5	8.3	3.9	9.3	6.6
Chelated Fe	10	18.0	11.5	8.0	3.9	8.7	7.3
	20	21.0	11.3	7.7	7.2	9.1	6.6
	40	17.5	11.7	8.7	5.5	7.9	7.0

^≠^Means under each column separated by the same lowercase letter (x, y, and z) were not significantly different among the mean values of different iron sources at p≤0.05.

**Fig 2:**
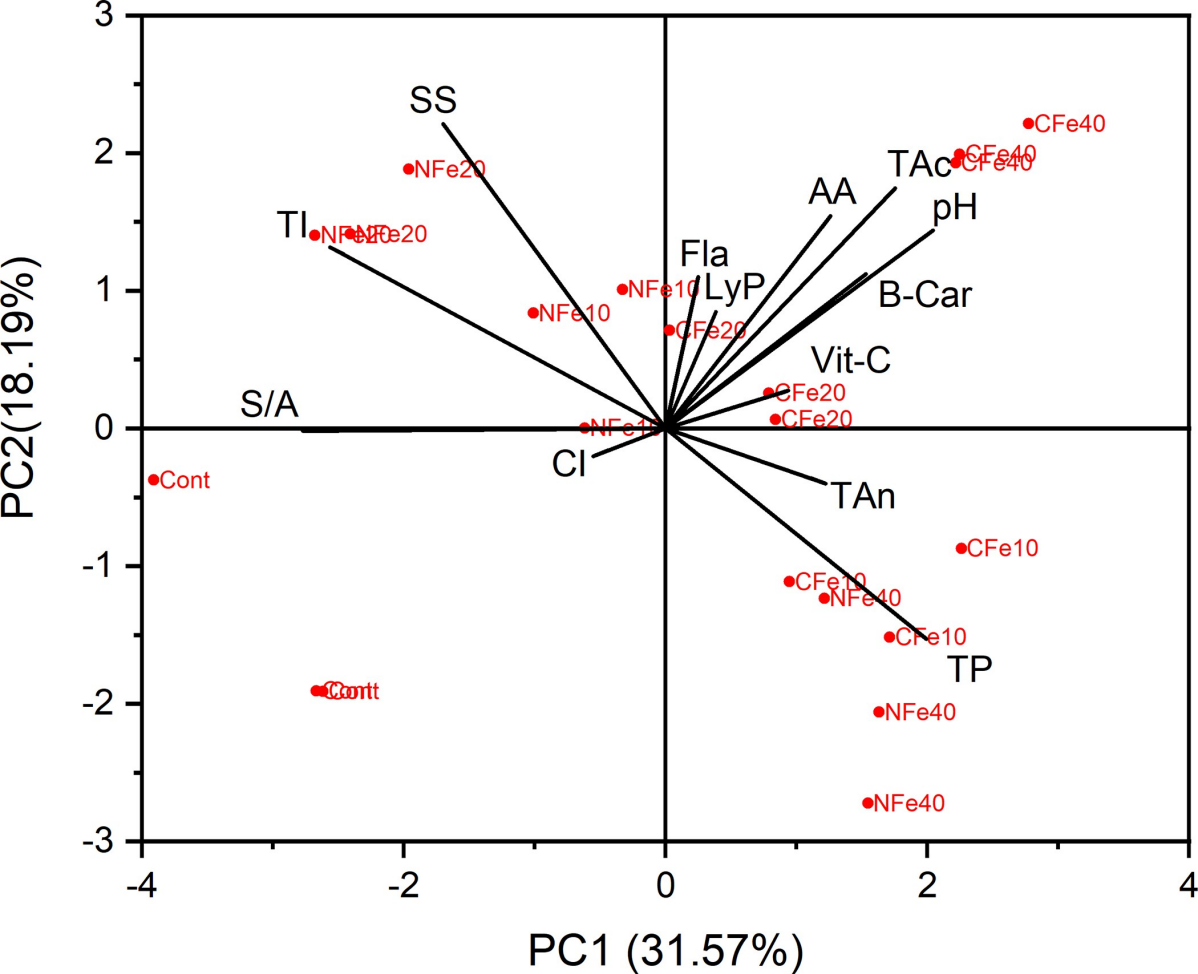
Principal components analyses to ascertain the effects of nano and chelated Fe fertilization on antioxidant properties of tomatoes (CI: Color index; TAc: Titratable acidity; SS: Soluble solids; LyP: Lycopene; Vit-C: Vitamin C; BCar: ß-carotenoid; Fla: Flavonoids; TP: Total polyphenols; TAn: Total anthocyanins; AA: Antioxidant activity or capacity; S/A: Sugar: acid; and TI: Taste index, NFe: Nano Fe; CFe: Chelated Fe; Cont: Control).

Using the PCA, the vitamin C, lycopene, and anthocyanin were identified as the core indicators among all the measured antioxidants to calculate the *NQ*_*Index*_ (*PCA-NQ*_*Index*_) of tomatoes in response to Fe fertilization (**Table 6; Fig 2**). The weights of the individual PCs were 0.59 (PC1) for vitamin C, 0.57 (PC2) for lycopene, and 0.71 (PC3) for anthocyanin (**Table 6**).

**Table 6:** Principal components analyses to select attributes for calculation of nutritional quality index based on tomato antioxidant concentration in response to different rates of nano and chelated Fe fertilization under a drip-irrigated plasticulture system.

Antioxidants	PC1	PC2	PC3
Eigenvalue	1.93	1.16	1.10
Variance (%)	32.2	19.3	18.3
Cumulative variance	32.2	51.5	69.8
Weight	0.46	0.28	0.26
**Factor loading**			
Vitamin-C	**0.59**	0.13	-0.35
Lycopene	-0.32	**0.57**	-0.31
β-carotene	0.001	0.74	0.44
Flavonoid	0.51	0.31	-0.27
Polyphenol	0.43	-0.09	0.03
Anthocyanin	0.32	-0.03	**0.71**

### Correlations among tomato properties associated with iron sources and rates

**Fig 3:**
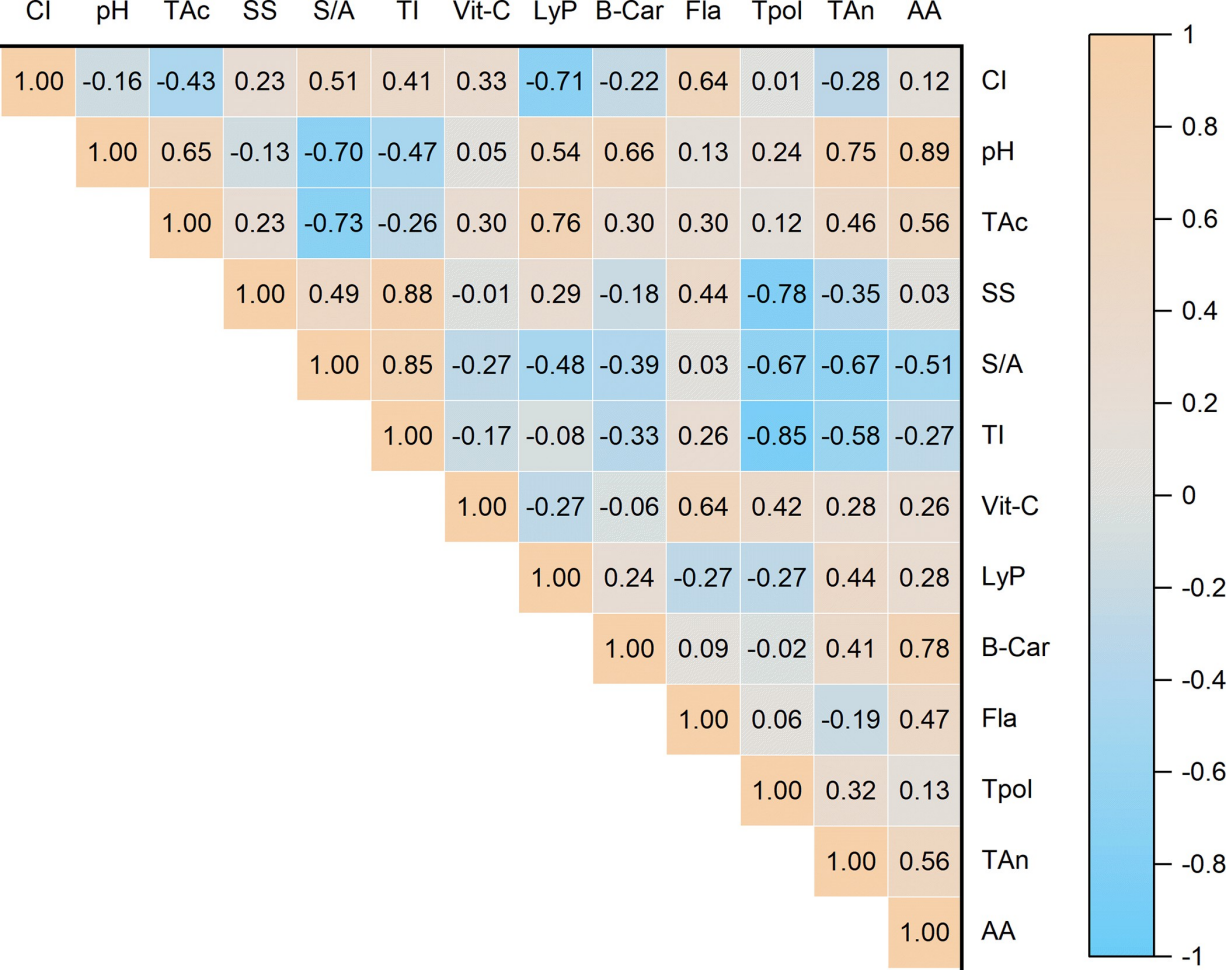
Correlation of antioxidant activity with measured phytochemical parameters in tomato fruits (CI: Color index; TAc: Titratable acidity; SS: Soluble solids; LyP: Lycopene; Vit-C: Vitamin C; BCar: ß-carotenoid; Fla: Flavonoids; TP: Total polyphenols; TAn: Total anthocyanins; AA: Antioxidant activity or capacity; S/A: Sugar: acid; and TI: Taste index). * Indicates significance at p≤0.05.

Pearson correlation coefficients were used to ascertain the effects of phytochemical parameters on the antioxidant activity of tomatoes (**Fig 3**). Results implied that the pH, titratable acidity, soluble solids, vitamin C, lycopene, β-carotene, flavonoid, polyphenol, and anthocyanin had a positive correlation with tomato antioxidant activities. While significant correlations were observed between antioxidant activity with pH (0.89), vitamin C (0.26), lycopene (0.28), and β-carotene (0.78), inverse relationships were observed between antioxidant activity with sugar:acid (-0.51) and taste index (-0.27).

The important antioxidant activity controlling parameters were highly influenced by 10, 20, and 40 mg/L chelated Fe treatments. Among them, chelated Fe (at 40 mg/L) treatment impacted most pronouncedly the titratable acidity, pH, and β-carotene of tomatoes. While lycopene and vitamin C concentration were strongly associated with the 20 mg/L chelated Fe treatment, the flavonoid concentration was influenced by the 10 mg/L nano Fe treatment. The taste index and soluble solids were highly associated with 20 mg/L nano Fe treatment.

**Fig 4:**
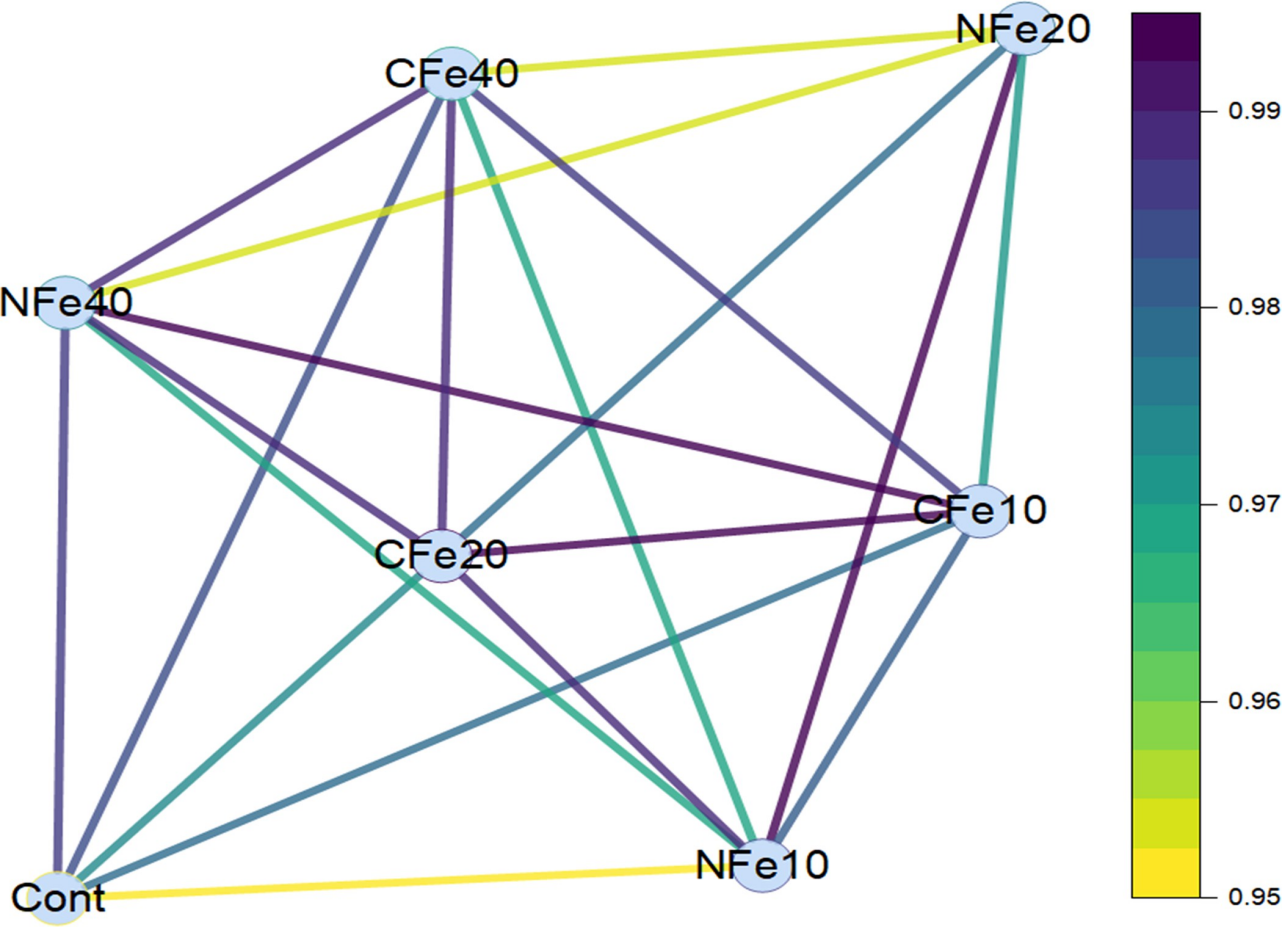
Correlation network among the different sources and rates of nano and chelated Fe fertilization based on phytochemical concentrations in tomato fruits [Cont: Control; NFe10: Nano Fe (10 mg/L); NFe10: Nano Fe (20 mg/L); NFe40: Nano Fe (40 mg/L); CFe10: Chelated Fe (10 mg/L); CFe10: Chelated Fe (20 mg/L); and CFe40: Chelated Fe (40 mg/L)].

To compare the effects of Fe sources and rates, a correlation network diagram was prepared based on the measured phytochemical properties of tomatoes (**Fig 4)**. Except for the nano Fe (20 and 40 mg/L) treatments, all the Fe treatments were highly correlated to each other (≥0.99). Nano Fe, especially 10 and 40 mg/L treatments, did not correlate significantly with other Fe treatments that correlate ≥ 0.99. It showed that chelated Fe (10 and 40 mg/L) treatments were highly correlated (~1.0), implying that both had a similar influence on controlling the physico-chemical and antioxidant properties of tomatoes. The control exhibited a correlation (<0.994) with the nano Fe (10 mg/L) treatment, but a high correlation with nano (40 mg/L) and chelated Fe (40 mg/L) treatments. This pattern suggests that both the control and the Fe treatments could produce similar physicochemical properties along with phytochemical concentrations associated with the antioxidant capacity of tomatoes. Conversely, both nano (40 mg/L) and chelated Fe (10 mg/L) treatments had a comparable influence on the phytochemical concentration of tomatoes. Among the different Fe sources and their rates, chelated Fe, when applied at 20 mg/L, demonstrated the highest correlation with the phytochemicals associated with the antioxidant capacity of tomatoes, making it the most effective treatment under these conditions.

### Fe fertilized tomatoes and public health benefits

Public health benefits are associated with the intake of antioxidants via adequate consumption of fruits and vegetables. The amount of antioxidant intake was calculated using 23.36 g/day/person of tomatoes consumed by humans (**Table 7**). A daily intake of 23.36 g of tomatoes, which have been fertilized with chelated Fe, provides 1.5 times higher vitamin C and 1.2 times higher polyphenol and flavonoid antioxidants compared to tomatoes fertilized with nano Fe. By consuming 23.36 g/day (0.05 lbs.) of either nano or chelated Fe-fertilized tomatoes, the same amount of lycopene and total anthocyanin would be provided; however, consumption of chelated Fe-fertilized tomatoes would provide a lower amount of β-carotene. As a whole, chelated Fe-fertilized tomatoes are expected to provide a higher amount of antioxidants compared to nano Fe-fertilized tomatoes.

**Table 7:** Antioxidant intake by a person consuming 23.36 g (0.05 lbs.) of nano or chelated Fe fertilized tomatoes per day [23.36 g or 0.05 lb. tomato intakes/day/person in the United States] [39].

Iron	Rate	Vitamin-C	Lycopene	β-carotene	Polyphenol	Flavonoid	Anthocyanin	Antioxidant capacity
source	(mg/L)			(mg/ 100 g)			(mg/g)	(μM/g)
	
					mg/day			
	
Control		3.9y≠	1.46x	0.12y	8.3y	0.43y	0.10x	36.2y
Nano Fe		3.1y	1.47x	0.16x	8.3y	0.44y	0.11x	37.9y
Chelated Fe		4.6x	1.47x	0.15x	9.7x	0.53x	0.11x	39.2x
Fe source x rate								
Nano Fe	10	3.9	1.46	0.18	7.7	0.51	0.11	36.8
	20	2.6	1.48	0.15	6.7	0.46	0.11	38.7
	40	2.8	1.47	0.16	10.5	0.37	0.11	38.2
Chelated Fe	10	4.4	1.47	0.15	9.9	0.38	0.12	38.7
	20	5.2	1.45	0.14	10.3	0.69	0.11	39.4
	40	4.3	1.49	0.16	8.9	0.52	0.11	39.6

≠Means under each column separated by the same lowercase letter (x, y, and z) were not significantly different among the mean values of different iron sources at p<0.05. AC: Antioxidant capacity

## Discussion

### Growth, yield, and physicochemical properties of tomato

Tomato plants under both nano and chelated Fe fertilization, when applied at 10 and 20 mg/L, showed significantly higher leaf SPAD values compared to the control. This increase was due to the effects of greater availability and uptake of Fe for leaf chlorophyll synthesis [40, 41]. Higher leaf SPAD values under nano Fe than under chelated Fe were associated with its greater stability, increased density of specific surface area, and enhanced reactivity to improve Fe availability for uptake by the tomato plants [17]. A consistently higher marketable yield and harvest index of tomato under nano Fe followed by chelated Fe, especially at 10 and 20 mg/L, when compared to the control was associated with increased nutrient-use efficiency to support chlorophyll synthesis with higher photosynthetic activity, followed by greater translocation of photosynthates to tomato fruits. It is expected that nano-sized particles may have a higher capacity of transport and faster delivery of nutrients via root channels to facilitate Fe uptake and its penetration deep inside the roots, leading to improved nutrient- and water-use efficiency of plants. Several studies have reported that nano fertilizers lead to higher productivity (6–17%) of vegetable crops, including tomatoes [31, 42]. Our results collaborated well with the results of previous studies, which reported that Fe fertilization improved tomato production [9, 43].

While the tomato color was related to the presence of lycopene (red color) and β-carotene (orange color) concentrations [44], a significant decrease in tomato color index under higher rates of Fe fertilization was associated with the excessive Fe uptake by the plants with an unbalanced nutrient-use efficiency to synthesize colored compounds. Our results on tomato juice pH were acidic and well-collaborated with the cited literature values that were below 4.5 [45]. The acidic pH is highly desirable because it controls microbial proliferation from spoilage of ripened tomatoes. A significantly higher titratable acidity under the chelated Fe fertilization than that of the nano Fe and the control was associated with the higher concentration of organic acids produced in tomatoes. This is also associated with slower rates of hydrolysis [45, 46]. A close relationship between the concentration of soluble solids (reducing sugars) and the pH of the tomato fruits suggests that tomatoes with higher sugar concentrations have less free H^+^ concentrations to produce acidity.

While sugars and organic acids are the major components of the soluble solids associated with the taste, sweetness, and flavor of tomatoes [47], glucose and fructose are the largest contributors to the soluble solids. Significantly lower sugar concentration (by 4%) in tomatoes, in response to both nano and chelated Fe fertilization, was due to the effects of slower rates of carbohydrate hydrolysis when compared to the control. One possible reason could be that both nano and chelated Fe treatments may have influenced the enzymatic activities related to carbohydrate metabolism in the plants. Fe is a key micronutrient in plant metabolic processes, including those involved in the production and functioning of enzymes. Accordingly, our results on sugar concentration were within the reported sugar values (4 to 6°Brix) for soluble solids in tomato fruits [48]. This indicated that the control treatment tomatoes had better flavor and taste than nano and chelated Fe-treated tomatoes, as higher sugar: acid controls the flavor and taste of the tomatoes [49].

### Antioxidant properties and nutritional quality of tomato

Significantly higher concentrations of antioxidants in response to the chelated Fe, followed by the nano Fe fertilization compared to the control, were associated with the higher leaf chlorophyll synthesis, which over time may have degraded, synthesized, and translocated as diverse organic compounds to the ripened tomatoes [50]. Vitamin C is one of the most important water-soluble antioxidants whose concentration increased (by 34%) by the chelated Fe was due to the synergistic effects of N-enriched chelated Fe on enzyme activities or vice-versa [51]. Further studies are needed to elaborate on the possible effects. Moreover, our results on vitamin C concentration in response to Fe fertilization ranged between 14.4 to 22.1 mg/100g, and were collaborated with the reported results in previous studies [52]. Likewise, significantly higher concentrations of carotenes with both chelated and nano Fe fertilization, especially with 20 mg/L, were due to the positive effects of Fe uptake optimization with increased chlorophyll synthesis [53]. It is reported that a transition from chloroplasts to chromoplasts formation during the ripening of tomatoes was associated with chlorophyll degradation and carotenoid synthesis [50].

A significantly higher carotene concentration with the cheated Fe compared to both nano Fe and the control treatments was due to the complementary effects of nitrogen associated with the N-enriched chelated compounds [52]. Nitrogen is the most critical macronutrient that forms the acetyl-CoA enzyme, which plays a critical role in the synthesis of carotenoids [54]. Our results on increased flavonoid concentration by the effects of chelated Fe fertilization were collaborated with the results of previous studies on Fe fertilization of tomatoes [42, 55]. A lack of significant difference in anthocyanin concentrations has indicated that Fe fertilization may not be directly linked to anthocyanin production. It is known that anthocyanins in plants are typically produced in response to abiotic and biotic stresses [56]. While several studies have reported that Fe fertilization does not significantly influence the lycopene, soluble phenolics, and flavonoids metabolisms [42], our results on tomato carotene, flavonoid, polyphenol, and anthocyanin concentrations in response to Fe fertilization, especially chelated Fe, were in line with the findings of other studies [57–60]. A significant increase in concentration of antioxidant compounds by Fe fertilization, especially chelated Fe, invariably increased the antioxidant capacity of tomatoes. Our results on the antioxidant capacity of tomatoes were in close agreement with the results of previous studies [34].

The PCA-based NQ_Index_ values were slightly lower, but followed a similar trend to the NQ_Index_ values as reported in other studies [32]. The higher nutrient-use efficiency of crops, as influenced by both chelated and nano Fe fertilization, might have led to improved nutritional quality of tomatoes [31, 42]. Based on our results, it is expected that the PCA-based method can be used to evaluate the nutritional quality of tomatoes based on core indicators of antioxidant compounds without analyzing or including a large number of variables that are complex, expensive, and time-consuming.

### Correlation matrix among the iron sources and rates and their effects on tomato properties

Results showed that chelated Fe (10 and 40 mg/L) treatments were highly correlated (~1.0), implying that both Fe levels had a similar influence on controlling the physico-chemical and antioxidant properties of tomatoes. A moderate correlation (<0.994) between the control and the nano Fe (10 mg/L) treatment, but a high correlation with both nano and chelated Fe (40 mg/L) treatments, suggested that both the control and Fe treatments would produce similar physicochemical properties along with phytochemicals related to the antioxidant capacity of tomatoes. Conversely, both the nano (40 mg/L) and chelated Fe (10 mg/L) treatments had a comparable impact on Fe fertilization on the phytochemical concentration of tomatoes. Across the Fe sources and rates, chelated Fe, when applied at 20 mg/L, demonstrated the highest correlation with the phytochemicals responsible for improving the antioxidant capacity of tomatoes, making it the most effective Fe treatment under a drip-irrigated plasticulture system.

### Iron fertilized tomatoes and public health benefits

The antioxidant capacity (Trolox equivalent) of fruits and vegetables is important in relation to oxygen radical absorbance capacity associated with public health. Tomatoes are believed to enhance the antioxidant capacity of humans when consumed as a routine part of their daily diet. It is reported that the human body produces free radicals and oxidative stress (25%) within their cells when inefficiently utilizing the carbohydrates by the mitochondria, which were unable to convert into adenosine triphosphates [61]. Therefore, 3,200 μM Trolox equivalent of antioxidant capacity provided by fruits or vegetables is required per serving to prevent a postprandial oxidative stress situation [62]. When an individual consumes 2,500 kcal of food/day, that person requires 11,500 μM Trolox equivalent antioxidant activity. An intake of 10,000 μM Trolox equivalent per day containing antioxidant-enriched food is expected to reduce the risk of hypertension, cerebral infarction, all-cause mortality, stroke, and endometrial cancer [63].

In our study, it was calculated that the consumption of only 23.36 g of chelated Fe-fertilized tomatoes will provide 39.7 μM Trolox equivalent antioxidant capacity, which is 1.4% and 8.4% higher than the antioxidant capacity of nano Fe-fertilized and control treatment tomatoes, respectively. However, only 0.39% of the total required antioxidants can be provided by the intake of 23.36 g of tomatoes per day, especially those fertilized with chelated Fe, which is insufficient to support a healthier life. This deficiency can be prevented by consuming more tomatoes, fruits, and other vegetables enriched in antioxidants when fertilized with Fe.

### Conclusions

Iron fertilization, especially 10 and 20 mg of nano or chelated Fe/L, significantly increased the marketable yield and harvest index of tomatoes. Chelated Fe when applied at 20 mg/L, significantly increased the vitamin C, carotene, flavonoid, and polyphenol concentrations than all other Fe treatments. Both chelated and nano Fe, when applied at 20 mg/L, had a similar antioxidant capacity to tomatoes. A higher nutritional quality index (NQ_Index_) of tomatoes was achieved with 20 mg/L chelated Fe; however, all the Fe treatments produced medium-level NQ_Index_ (<70–40) values. By using principal components analyses, vitamin C, lycopene, and anthocyanin were identified as the orthogonal core indicators to calculate the PCA-NQ_Index_, which ranged between 47 to 54 and were within the range of medium-level NQ_Index_. Among the Fe sources and rates, chelated Fe, when applied at 20 mg/L, was the most appropriate rate associated with the improved antioxidant capacity of tomatoes.
